# Recommendations on complementary and alternative medicine within S3 guidelines in oncology: systematic quality assessment of underlying methodology

**DOI:** 10.1007/s00432-020-03238-2

**Published:** 2020-05-11

**Authors:** Sabine Kutschan, Maren Freuding, Christian Keinki, Jutta Huebner

**Affiliations:** Klinik für Innere Medizin II, Hämatologie und Internistische Onkologie, Universitätsklinikum Jena, Am Klinikum 1, 07747 Jena, Germany

**Keywords:** Complementary medicine, Alternative medicine, Cancer, Guidelines, Evidence-based medicine

## Abstract

**Purpose:**

Complementary and alternative medicine (CAM) is used by about half of all patients with cancer. Guidelines are an important tool to introduce evidence-based medicine into routine cancer care. The aim of our study was to assess methodology of the statements and recommendations concerning CAM.

**Methods:**

A systematic assessment of all S3 guidelines published until November 2018 was done. Methodology of all statements and recommendations concerning CAM which were declared as evidence-based was evaluated with respect to international standards. According to the AMSTAR-2 instrument search strategy including filters, searched databases, restrictions to the research question and description of the included studies were examined. In case of adaptations from other guidelines, all underlying guidelines were examined as well.

**Results:**

After examining 212 guidelines, 82 evidence-based statements and recommendations regarding CAM could be identified. Four were derived by adaptation, 78 by a de-novo search. Only 11 of 78 (14%) fulfilled all assessment criteria. In 18 (19%) cases no information on search strategy was attainable in any document affiliated to the guideline, in 35 (45%) cases information on search strategy was superficial and in 54 (78%) cases the referred evidence was not presented in adequate detail.

**Conclusions:**

Concerning CAM statements and recommendations within S3 guidelines quality of evidence processing has several shortcomings. Guideline adaptions often lack transparency and traceability.

**Electronic supplementary material:**

The online version of this article (10.1007/s00432-020-03238-2) contains supplementary material, which is available to authorized users.

## Introduction

With a worldwide growing number of publications, evolving new study designs and statistical methods, guidelines become more important for treatment planning of service providers and information and decision-making of patients. Guidelines are an essential tool for promoting quality and transparency of medical care. High quality guidelines are systematically developed by independent institutions and scientific working groups around the world. The German Guideline Program in Oncology (GGPO), launched in cooperation by the Association of the Scientific Medical Societies in Germany (AMWF), the German Cancer Society (DKG) and the German Cancer Aid (DKH) in 2008, is the most important national program on cancer guidelines in Germany. The methodology of guideline development in this program is consistent with international standards (Qaseem et al. 2012) and follows the German national standards defined by the AWMF in particular. The methodological characteristics in the development of guidelines are defined by the stages S1–S3, whereas S3 refers to the highest quality. These guidelines are developed by a full formalized, systematic process. In a first step key questions are defined, for which a systematic search and assessment of the evidence is the base for statements and recommendations. In a second step these are revised and consented by representatives of involved scientific and professional societies. For each statement and recommendation the grade of evidence and the corresponding consensus or, in case of missing evidence, only the consensus is stated. Most of the guidelines in the GGPO are S3 guidelines. Also other international guideline programs live up to these standards, as those of the American College of Chest Physicians (CHEST), American Society of Clinical Oncology Journal (ASCO), Agency for Healthcare Research and Quality (AHRQ), American Society of Hematology (ASH), American Society for Radiation Oncology (ASTRO), American Urological Association (AUA), Mc Master University (CA), Belgian Healthcare Knowledge Center (KCE), Cancer Council Australia (CCA) and Dutch Institute for Healthcare Improvement (CBO). Besides guidelines discussing the complete treatment of different cancer entities cross-sectional guidelines exist, which focus on supportive therapy, psycho-social care, rehabilitation or complementary medicine in all cancer entities.

Complementary and alternative medicine (CAM) is used by about half of all patients with cancer (Horneber et al. 2011; Paul et al. 2017; Micke et al. 2009; Molassiotis et al. 2005). In patients with breast cancer user rate is up to 90% (Micke et al. 2009). Nevertheless, the evidence for many CAM-interventions is inconclusive due to a lack of high-quality studies (Huebner 2013). The reasons are manifold and involve less funding or the focus on conventional medicine in the daily routine of physicians. This lack of evidence leads not only to lost opportunities in the treatment of a patient group, which is suffering from a wide range of symptoms by the cancer itself and well-known side effects of treatment, but also to a risk of adverse events due to an direct effect or an interaction of complementary methods and substances with conventional cancer treatment. These risks are enhanced as most patients do not disclose their CAM usage to their oncologist (Robinson and McGrail 2004; Saxe et al. 2008).

A broader definition of CAM includes all methods decided and conducted by the patient himself. Accordingly, nutrition including supplements with vitamins and trace elements or cancer diets may be considered as CAM (Cassileth and Deng 2004; Vickers and Cassileth 2001). In this article, we will use this broader definition.

The aim of this study was to investigate how CAM-methods are presented in S3 guidelines of the mentioned guideline programs. The research questions are the following:

- How many CAM statements and recommendations are in the guidelines?
- How many and which of the CAM statements and recommendations are evidence-based and how many are only consensus based? Is this distribution comparable to recommendations for conventional medicine?
- How many of the evidence-based statements and recommendations really the standard criteria of the development for guidelines (as defined by the AWMF 2012)?

## Methods

All S3 guidelines published until November 2018 on the website of the GGPO (Leitlinienprogramm Onkologie Berlin 2019) were included in the review. Additionally the database of the Guidelines International Network (GIN) has been searched, using “oncolog*” and “cancer” as search terms without any further restrictions. All guidelines from the ASCO, AHRQ, ASTRO, KCE, ASH, CA, AUA, CHEST, CCA, CA and CBO published between 2012 and 2018 were retrieved.

All following steps have been conducted by two researchers (SK, MF) independently. Any deviation has been discussed and solved mutual. If necessary, a third expert was involved (JH). The first step was a systematic search of the following vocabulary and root words (here translated to English):

“alternative”, ”complementary”, “integrative”, “acupuncture”, “anthroposophy”, “autogenic training”, “biological”, “Chinese”, “enzyme”, “exercise”, “folate”, “homeopathy”, “hypnosis”, “massage”, “meditation”, “mind-body”, “movement”, “naturopathy”, “physiotherapy”, “phytotherapy”, “plants”, “qigong”, “relaxation”, “sports”, “tai chi”, “TCM”, “vitamins”, “yoga”.

Additionally all directories of the guidelines have been screened for any CAM-interventions. All relevant text passages were examined for statements and recommendations.

## Evaluation

All statements and recommendations in the considered guidelines that were declared as “evidence-based” were examined for their methodological quality. For evaluation items 4 and 8 of the AMSTAR 2 instrument (Shea et al. 2007a, b, 2009, 2017; Pieper et al. 2012) were used as follows:

1. [Item 4:] Did the review authors use a comprehensive literature search strategy?
  - Provide a search strategy or at least key words with comprehensive vocabulary?
  - Search at least two databases?
  - Use appropriate restrictions to the research question (inclusion/exclusion criteria, publication type, study type)?
2. [Item 8:] Did the review authors describe the included studies in adequate detail? (Are evidence tables presented?)

These key points aim directly at the underlying research of the individual statement or recommendation. Since different searches are usually carried out for different PICO questions, the methodology can be different for each PICO- question of one guideline. The other items of the AMSTAR 2 relate to the guideline as a whole and were therefore not adequate to our research question. The focus of the present work lies exclusively on the parts of a guideline concerning CAM medicine.

## Results

A total of 212 German and international guidelines were retrieved and examined. Altogether 82 evidence-based recommendations or statements on CAM were found in the following 18 guidelines:

- ESPEN Guidelines on Enteral Nutrition: Surgery including Organ Transplantation (Weimann 2006; Schütz 2006).
- DGEM Guidelines on enteral nutrition (Lochs and Weimann 2003; Lochs and Krys 2004).
- ACS Nutrition and physical activity guidelines for cancer survivors (Rock 2012).
- US Department of Health and Human Services Physical Activity Guidelines for Americans (Services UDoHaH 2008).
- ACS Nutrition and physical activity during and after cancer treatment (Doyle 2006).
- ACS Nutrition during and after cancer treatment (Brown et al. 2001).
- ACS Nutrition and physical activity during and after cancer treatment (Brown et al. 2003).
- ACS Guidelines on diet, nutrition, and cancer prevention (ACS 1996).
- ACS guidelines on diet, nutrition, and cancer (Weinhouse et al. 1991).
- ACS Clinical Practice Guidelines on the Evidence-Based Use of Integrative Therapies During and After Breast Cancer Treatment (Heather Greenlee et al. 2017).
- ACS/ ASCO Breast Cancer Survivorship Care Guideline (Runowicz et al. 2016).
- ACS/ ASCO Breast Cancer Survivorship Care Guideline (Carolyn et al. 2016).
- ASCO Prevention and Management of Chemotherapy-Induced Peripheral Neuropathy in Survivors of Adult Cancers (Dawn et al. 2014).
- ASCO Management of Chronic Pain in Survivors of Adult Cancers (Judith et al. 2016).
- ASCO Antiemetics (Hesketh et al. 2017).
- ASCO Prostate Cancer Survivorship Care Guideline (Matthew et al. 2015a, b).
- CHEST Chemoprevention of Lung Cancer (Eva Szabo, et al. 2013).
- CHEST Symptom Management in Patients With Lung Cancer (Michael et al. 2013).
- CHEST Complementary Therapies and Integrative Medicine in Lung Cancer (Gary et al. 2013).
- DVO Guidelines on Osteoporosis in men over the age of 60 and in postmenopausal women (Dachverband Osteologie e.V. 2014).
- GPO Early detection, diagnosis, therapy and follow-up of the urinary bladder carcinoma (Margitta Retz 2016).
- GGPO Prevention, diagnosis, therapy and follow-up of lung cancer (Dieter Ukena 2018).
- GGPO Diagnosis and treatment of adenocarcinomas of the stomach and esophagogastric junction (Möhler et al. 2012).
- GGPO Diagnosis, therapy and follow-up of melanoma (Thomas Eigentler et al. 2018).
- GGPO Psycho-oncological diagnostics, counseling and treatment of adult cancer patients (Adolph et al. 2014).
- GGPO Supportive therapy for oncological patients (Karin Jordan et al. 2017).
- KCE Supportive Treatment for Cancer, Part 2: Prevention and Treatment of Adverse Events related to Chemotherapy and Radiotherapy (Leen Verleye et al. 2012).
- PEBC/CCO Follow-up Care and Psychosocial Needs of Survivors of Prostate Cancer (Matthew et al. 2015a, b).

Four (5%) of the evidence-based statements and recommendations were adapted from other guidelines and 78 (95%) were based on de-novo searches by the guideline authors. The results of the methodological examination of all 82 evidence-based CAM- statements and recommendations are presented in detail in the supplement. An overview is presented in Table 1.

**Table 1:** Results of methodological examination (according to criteria of AMSTAR-2 instrument) of all evidence-based recommendations on CAM

Criteria: Did the research authors...	Rating	Number of cases (%)
1: ...Provide a search strategy or at least key words with comprehensive vocabulary?	Yes: detailed information on search strategy	25 (32%)
	Partly: superficial information on search strategy	35 (45%)
	No: no information on search strategy	18 (23%)
2: Search at least 2 databases?	Yes: at least two databases	59 (76%)
	Partly: only one database	0 (0%)
	No: no information on databases	19 (24%)
3: Use appropriate restrictions to the research question (inclusion/exclusion criteria, publication type, study type)?	Yes: adequate restrictions of search strategy	20 (26%)
	Partly: narrow search vocabulary	35 (45%)
	No: clearly inadequate restrictions of search strategy	23 (29%)
4: Describe the included studies in adequate detail/presented evidence tables?	Yes: detailed presentation of referred evidence	24 (31%)
	Partly: some evidence is presented in detail or evidence tables are lack important information as	27 (35%)
	No: none of the referred evidence is presented in detail	27 (35%)
All criteria 1–4	Completely fulfilled: all criteria are YES	11 (14%)
	Almost fulfilled: all criteria are YES, but one criteria is PARTLY	8 (10%)
	Partly fulfilled: two or more criteria are PARTLY, all others are YES	26 (33%)
	Not fulfilled: one or more criteria are NO	33 (42%)

## Evaluation of de-novo searches

Altogether, in only 25 of the 78 statements and recommendations (32%) derived from de-novo searches detailed information on the search strategy could be found. In 35 cases (45%) only superficial information and in 18 cases (23%) no information on the search strategy was attainable in any publication affiliated to the guideline. In 59 cases (76%) the search was performed on at least two databases. There was no case of performing the search on only one database (0%), but in 19 (24%) cases no information about the used databases was given. The search restrictions were adequate in 20 cases (26%).They were only partly adequate in 35 cases (45%), because vocabulary of search terms was too narrow, recommendations exceeded the searched topic or selection of study types was not conclusive. In 23 cases (29%) search restrictions were clearly inadequate and for 24 of the 78 statements and recommendations (31%) the referred evidence was presented in detail, meaning evidence tables or comparable systematic presentations of relevant details of the included studies were displayed. In 27 cases (35%) evidence tables lacked important information on e.g. study population characteristics, study size or concrete results. In 27 cases (35%) no detailed information on included studies was found, furthermore an assessment of the bias risk of included studies was missing as well.

Only eleven of the 78 statements and recommendations (14%) fulfilled all the assessment criteria. Eight further statements (10%) met the criteria almost completely (one of the four criteria was only fulfilled partly), 26 cases (33%) fulfilled all criteria in general only partly and 33 cases (42%) failed to fulfill the criteria.

## Evaluation of guideline adaptations

Adaptations of CAM statements or recommendations took place in four different guidelines [on stomach cancer by GGPO (Möhler et al. 2012), breast cancer by GGPO (Achim Wöckel and Janni 2018), prostate cancer survivorship by ASCO (Matthew et al. 2015a, b) and supportive therapy by GGPO (Karin Jordan et al. 2017)], meaning one or more other guidelines were consulted. In the process of adaptation an updating search by applying the search strategy from the other guideline should be done, if the search of the underlying guideline is expected to be outdated. In three cases an updating search was clearly not performed.

In the first case concerning a recommendation on immunomodulatory energy supplements before major tumor resection [guideline on adenocarcinoma of the stomach (42: recommendation number 118)], the adapted guideline was six years old (Weimann 2006; Schütz 2006). It was written that a comprehensive literature search was carried out, but neither the search strategy nor the included studies were presented in detail.

In the second case of a guideline adaptation [guideline on breast cancer (48: recommendation numbers 4.75–4.79, 6.47)] a recommendation concerning nutrition was derived from a guideline which was only two years old (Runowicz 2016), but the contents of that underlying guideline were also derived from a series of other guideline adaptations and updates. Overall nine different versions and updates were found (details are in the supplement). In none of these underlying guidelines a detailed search strategy could be found. Likewise the referred evidence was not presented in detail in any of these guidelines.

In the third case, concerning a recommendation on vitamin D to prevent osteoporosis in any cancer type [guideline on supportive therapy (45: recommendation number 10.54)], the adapted guideline considered only men and woman more than 60 years old and the original statement restricted the recommendation of vitamin D supplementation to people at high risk of falling or fractures with low exposure to sunlight (Dachverband Osteologie e.V. 2014). This restriction was not repeated in the recommendation. Moreover, the underlying guideline had a very short methodology report, showing no details about the search strategy and the included evidence. In addition, the search was performed in only one database (Medline).

The last case concerning a recommendation on diet with supplementation of vitamin D for prostate cancer survivors (Matthew et al. 2015a, b), according to the authors the search strategy from another guideline was adapted and performed at one database (Medline), but the supplement was not available and no further information could be retrieved. The adapted guideline also searched only one database (Medline), presented only general search terms, made incomprehensible restrictions and missed to supply evidence tables or detailed information on included studies.

## Discussion

Complementary medicine is becoming a more and more important issue for patients with cancer. On the other hand, these topics are not sufficiently taken into account in medical education and quality-assured information is difficult to find for physicians and patients as well. For that reason, inclusion of recommendations regarding CAM in national guidelines is important as they provide orientation, evidence-based information and help to increase benefit and reduce risks of these methods.

But, as the work of Huebner and Follmann (2013) shows, the level of evidence in the guidelines of the GGPO differs between conventional medicine and CAM. For conventional therapy about a third of recommendations based on level 1 evidence, another third on level 2 and 3 and the rest on level 4, 5 or good clinical practice (GCP). For CAM, this ratio is different with two thirds being level 4, 5 or GCP and only a quarter being level (Huebner and Follmann 2013). For any user of guidelines, transparency on the evidence of the statements is highly important. In the field of CAM usually low level evidence is available, consecutively only weak recommendations can be made in guidelines. Moreover, there has been an ongoing discussion on the validity of the concept of evidence-based medicine for holistic methods as homeopathy or anthroposophic medicine. Some proponents of these methods prefer the concept of empirical medicine as a collective knowledge or cognition based medicine, as the knowledge of the individual physician, derived from his professional formation and experience (Kiene 2005; Raspe 2005). On the other hand, the number of clinical studies, randomized controlled studies and systematic reviews in CAM is rising, thus providing more and more data. Yet, the work-up of these data is difficult, as the quality of many studies in this field is low. Moreover, systematic reviews also are heterogeneous in quality (Jutta Huebner and Muecke 2013).

We don’t want to criticize the heterogeneity of systematic reviews that is caused by heterogenic or sparse results from studies. And we don’t criticize the use of consensus-based recommendations. These concepts are useful to reflect and discuss the actual standard of care. Experts can also have an insight based on experiences and non-published scientific data. And also due to the high demand from patients, several guidelines decided to use also consensus-based recommendations, for example the guidelines of the German Society of Palliative Care (Palliativmedizin 2015). These recommendations may help physicians and nurses to guide patients safely through CAM, bridging the time until studies of high quality have been conducted. However, our data show that the quality of evidence processing in German and international guidelines with respect to CAM has several shortcomings. In many cases it is very difficult to understand the methodology behind a statement or recommendation. There is a lack of high quality systematic searches and presentation of the evidence.

In case of adaptations from former (international) guidelines, transparency and traceability are often lacking. In one case we had to search nine different versions of a guideline and other underlying guidelines to get information about the search strategy and the included evidence, which in the end were not described in detail anywhere. To deal responsibly with time and financial resources and to enable timely updates of guidelines, it is necessary to adapt previous guidelines and to refer to systematic reviews. However, the process of adaptation may lead to methodological faults being handed on from one guideline to another. Our suggestion is that in a guideline adaptation the methodology of the underlying guideline should not only be evaluated (e.g. with the meanwhile established DELBI procedure), but also the exact details of the methodology (concrete search strategy, time frame, restrictions of search) should be repeated. If no details on the search strategy can be found, a de-novo search should be done.

## Electronic supplementary material

Below is the link to the electronic supplementary material.

Supplementary material 1 (DOCX 67 kb)
