# Supplementary material for: Recommendations on complementary and alternative medicine within S3 guidelines in oncology: systematic quality assessment of underlying methodology
Source: J Cancer Res Clin Oncol. 2020 May 11;146(9):2419–25. doi: 10.1007/s00432-020-03238-2 (PMC7382657; doi:10.1007/s00432-020-03238-2)
Supplement: Supplementary file 1 — Supplementary material 1 (DOCX 67 kb) [file 432_2020_3238_MOESM1_ESM.docx]

| **Guideline** | **Keywords** | **Statement (Number of Statement/Recommendation: Page)** | **Evaluation*** |
| --- | --- | --- | --- |
| **Statements and recommendations relying on a de-novo search** | | | |
| Urinary bladder Carcinoma [Harnblasenkarzinom] – GGPO- Version 1.1 - November  2016 | Hyperthermia | “The adjuvant intravesical mitomycin-C-treatment under conditions of microwave-induced hyperthermia is an experimental procedure and it should be applied only in the context of prospective studies.” (6.37: p. 127) | 1. Yes: search strategy is presented in the report (p. 194) 2. No: only Medline 3. Yes: non-invasive carcinoma of the urinary bladder with different spellings, 2003-20016, meta-analyses, systematic reviews and randomized controlled trials (using the default filters on Medline) 4. Yes: The studies are presented in detail in evidence tables. |
| Lung carcinoma [Lungenkarzinom] – GGPO- Version 1.0, February 2018 | Complementary Medicine  Diet  Nutrition  Relaxation | “Patients who are or have been diagnosed with lung cancer should be actively asked about the use of ‘complementary medicine’ medicines and procedures. They should be given the opportunity to receive reliable information and expert advice on this subject in an open and needs-based conversation.” (13.1: p. 327)  “The use of substances where proven efficacy on survival, tumor response or quality of life and associated factors is not demonstrated by meaningful studies is not recommended outside of clinical trials.” (13.2: p. 327)  “When taking herbal preparations, it should be clarified whether pharmacodynamic or pharmacokinetic interactions with concomitantly used conventional drugs or diagnostics are possible.” (13.4: p. 327)  “During chemo- or radiotherapy, dietary supplements with antioxidant effects should not be given in dosages that exceed the reference levels for daily use indicated by specialist companies in Germany, Austria and Switzerland” (13.4: p. 328)  “Patients who are or have had lung cancer should be offered expert nutritional counselling tailored to their medical condition, current therapy and needs“ (13.5: p. 328)  “Whenever possible, consideration should be given to the use of non - medical procedures to reduce discomfort or distress due to illness or disease.” (13.6: p. 328) | 1. No: No search strategy presented 2. No: – 3. No: – 4. No: – |
| Stomach cancer [Magenkarzinom] – GGPO – February 2012 | Mistletoe | „There are weak indications of an improvement in the quality of life of mixed populations of patients with different types of tumors.” (143: p. 145) | 1. No: No search strategy presented. According to the information, a de-novo search was carried out, but no further details can be found. 2. No: – 3. No: – 4. No: – |
|  | Homeopathy | “A therapeutic benefit of homeopathy is not proven in gastric carcinoma as a supportive therapy“ (146: p. 147) | 1. No: No search strategy presented. According to the information, a de-novo search was carried out, but no further details can be found. 2. No: – 3. No: – 4. No: – |
| Melanoma [Melanom] – GGPO – Version 3.1, July 2018 | Mistletoe | “Adjuvant therapy with mistletoe preparations should not be given.” (6.36: p. 102) | 1. Yes: Search strategy is presented in an extra document (p. 1197) 2. Yes: Primary search 2012: Medline, Cochrane library and Embase; Update 2016: Medline and Cochrane library 3. Partly: wide search on Medline including mesh terms, but on Cochrane and Embase only title and abstract fields are searched for ‘melanoma’ and ‘mistleloe’ or ‘viscum album’ 4. Yes: Studies are presented in detail in evidence tables in the extra document (p. 1199 ff) |
| Psycho-oncology [Psychoonkologie] – GGPO – Version 1.1, January 2014 | Relaxation | “Relaxation should be offered to people with cancer, regardless of stress level.” (8.6: p. 60) | 1. Yes: Search strategy is presented in a systematic review, published by the authors in the Journal of Clinical Oncology. Referral in the report (p. 101) 2. Yes: Medline, PsycInfo and Cochrane Central register of Controlled Trials 3. Partly: Restriction only on RCTs, no restrictions on cancer type, broad vocabulary of psychological interventions, but terms where entered in concrete spellings (e.g. only ‘hypnotherapy’, not ‘hypnosis’) in title, abstract and textword fields. 4. Yes: Studies are presented in detail in evidence tables in the publication of the Systematic Review   Annotation: The results of the different treatment methods were not evaluated separately. The following interventions were all subsumed under ‘Relaxation Procedures’: Progressive Muscle Relaxation (PMR) plus Imagery (16; 34%), PMR alone (14; 30%), Hypnosis (6; 13%), Imagery (4; 9%), Yoga or Meditation (3, 6%), PMR plus desensitization (2, 4%), and autogenic training (2%), desensitization (2%) and biofeedback (2%). |
|  | Massage  Yoga | “To relieve anxiety, massages can be performed in addition to yoga.” (9.9: p. 74) | 1. Yes: Search strategy is presented in the report (p. 201 ff) 2. Yes: Medline, PEDro, Cochrane Controlled trial register, Cinahl 3. Partly: Same search strategy that is used for exercise treatments. This strategy focusses on exercise and activity and doesn’t have any explicit yoga or massage terminology like ‘yoga’, ‘qigong’, ‘massage’, ‘shiatsu’ or ‘tuina’. Although ‘quality of life’ is included in the PICO-question, its vocabulary is only executed on PEDro and Cinahl. Restriction on publication years 2000-2014, no restrictions on cancer type. 4. Yes: Studies are presented in detail in evidence tables in the report (p. 214) |
| Supportive therapy [Supportive Therapie] – GGPO – Version 1.1, April 2017 | Acupuncture  Complementary and Alternative medicine  Homeopathy  Medicinal herbs | “Due to missing RCT, no recommendation for the use of complementary or alternative medicine (e.g., homeopathy, acupuncture, medicinal herbs, Schüssler salts, beetroot juice, Nettle juice, black cumin oil, red wine, amalgam removal, treatment of alleged interference fields on the teeth) is possible in tumor therapy-induced anemia.” (3.11: p. 65) | 1. No: According to the information a de-novo search was carried out, but the search strategy presented in the report does not include any CAM-treatments of anemia, but only the treatment with EPO, biosimilar or iron (p. 109ff). CAM-treatments (alternative, complementary, homeopathic, aloe vera, beta-carotene, chamomile, chinese herbal, folic acid, glutamine, hydrolytic enzyme, MF 5232 (mucotrol), multivitamin, natural, polaprezinc, traumeel, tretinoin, vitamin, zinc, honey, manuka & kanuka oil, Rhodiola algida, vitamin A, vitamin E, Wobe-Mugos E, retinoid, and indigo wood root) are only searched in compelling combination with oral mucositis due to systemic tumor therapy on Medline (p. 119 ff.). 2. – 3. – 4. – |
|  | Traditional Chinese Medicine TCM | Chapter on tumor therapy induced anemia: “Treatment effects from Traditional Chinese Medicine (TCM) studies cannot be assessed without bias due to translation issues, so no recommendation is possible.” (3.12: p. 65) | 1. Same as above. In addition, kampo was searched separately, but only in combination with tumor therapy-induced diarrhea on Central and Medline (p. 114) 2. – 3. – 4. – |
|  | Nutrition  Vitamins | On the topic prevention of oral mucositis due chemotherapy: “The following substances (topical or systemic) do not have sufficient evidence to give a recommendation for or against use: ... honey, chamomile, vitamin A, vitamin E, combination of vitamins ...” (7.8: p. 165) | 1. Yes: Search strategy is presented in the report (p. 119 ff) 2. No: Medline 3. Yes: Wide search terms like ‘alternative’ and ‘complementary’, as well as concrete treatments as ‘homeopathic’, ‘aloe vera’, ‘beta-carotene’, ‘chinese herbal’, ‘vitamin’ and others are searched in compelling combination with oral mucositis due to systemic tumor therapy, no restrictions on study or cancer type 4. No: None of the mentioned studies in presented in detail in evidence tables. |
|  | Acupuncture | On the prevention of radiogenic xerostomia:  “The prophylactic use of classical acupuncture therapy improves subjective and objective parameters of radiogenic xerostomia. It can be used.” (12.78: p. 412)  On the treatment of radiogenic xerostomia:  “Acupuncture can improve subjective parameters of xerostomia and can be used.” (12.83: p. 417) | 1. Yes: Search strategy is presented in the report (p. 135) 2. Yes: Central and Medline 3. Yes: Extensive search strategy for radiogenic xerostomia, no restriction on cancer or study types, ‘acupuncture’ was entered as MeSH term, as well as broad terms like ‘protective treatment’ or ‘supportive care’ 4. Yes: Studies are presented in detail in evidence tables (p. 479) |
| **International Guidelines** |  |  |  |
| Prevention and Management of Chemotherapy-Induced  Peripheral Neuropathy in Survivors of Adult Cancers-  ASCO - 2014 | Acetyl-L-carnitine (ALC) | “Clinicians should not offer the following agents for the prevention of CIPN to patients with cancer undergoing treatment with neurotoxic agents: Acetyl-L-carnitine (ALC)” (p. 1942) | 1. Yes: search strategy is presented online 2. Yes: Medline, Embase, Amed 3. Yes: broad search string; restrictions on adults, rcts, systematic reviews, meta-analyses were adequate 4. Yes: Studies are presented in detail in the evidence tables (Hershman 2013) |
|  | CaMg for patients receiving oxaliplatin-based chemotherapy | “Clinicians should not offer the following agents for the prevention of CIPN to patients with cancer undergoing treatment with neurotoxic agents: CaMg” (p. 1942) | 1. Yes: search strategy is presented online 2. Yes: Medline, Embase, Amed 3. Yes: broad search string; restrictions on adults, rcts, systematic reviews, meta-analyses were adequate 4. Yes: Studies are presented in detail in the evidence tables   (Ishibashi 2010, Chay 2010, Grothey 2011, Grothey 2008, Loprinzi 2013) |
|  | Glutathione (GSH) for patients receiving paclitaxel/carboplatin chemotherapy | “Clinicians should not offer the following agents for the prevention of CIPN to patients with cancer undergoing treatment with neurotoxic agents: Glutathione (GSH) for patients receiving paclitaxel/carboplatin chemotherapy” (p. 1942) | 1. Yes: search strategy is presented online 2. Yes: Medline, Embase, Amed 3. Yes: broad search string; restrictions on adults, rcts, systematic reviews, meta-analyses were adequate 4. Yes: Studies are presented in detail in the evidence tables   (E-tabelle zu Bogliun 1996, Cascinu 1995, Cascinu 2002, Leal 2013, Mila 2009, Schmidinger 2000, Smyth 1997) |
|  | Vitamin E | “Clinicians should not offer the following agents for the prevention of CIPN to patients with cancer undergoing treatment with neurotoxic agents: Vitamin E” (p. 1942) | 1. Yes: Search strategy is presented online 2. Yes: Medline, Embase, Amed 3. Yes: Broad search string; restrictions on adults, rcts, systematic reviews, meta-analyses were adequate 4. Yes: Studies are presented in detail in the evidence tables   E-tabelle zu Kottschade 2011, Argyriou 2006, Pace 2003, Pace 2010 |
| Supportive Treatment for Cancer, Part 2: Prevention and Treatment of Adverse Events related to Chemotherapy and Radiotherapy- KCE - 2012 | Zinc | Mucositis: “The use of […] zinc mouth washes can be considered to prevent oral mucositis in patients receiving chemotherapy and/or radiotherapy (weak recommendation)”. (p. 30) | 1. Yes: Search strategy is presented in appendix 2. Yes: Medline, PreMedline, Embase, Central 3. Yes: Broad vocabulary for cancer and mucositis or skin toxicities without restrictions on intervention types; restriction on rcts was adequate and transparent 4. Yes: The relevant study is presented in detail in an evidence table |
|  | Honey | Prevention of oral mucositis: “The use of honey can be considered to prevent oral mucositis in patients undergoing (chemo)radiotherapy (weak recommendation).” (p. 34)  Treamtent of oral mucositits: “Honey is not recommended to treat oral mucositis due to chemo- and/or radiotherapy (weak recommendation).” (p. 39)  Skin problems: “The use of honey gauze to treat radiodermatitis (weak recommendation).” (p. 51) | 1. Yes: Search strategy is presented in appendix 2. Yes: Medline, PreMedline, Embase, Central 3. Yes: Broad vocabulary for cancer and mucositis or skin toxicities without restrictions on intervention types (mucositis)/ with sufficient terms on intervention (skin toxicities); restriction on rcts was adequate and transparent 4. Yes: All studies (rcts and srs) are presented in detail in evidence tables |
|  | Foot soak | Skin toxicities: “There is insufficient evidence to recommend foot soaks to prevent or treat skin toxicity due to cancer treatment” (p.50) | 1. Yes: Search strategy is presented in appendix 2. Yes: Medline, PreMedline, Embase, Central 3. Partly: Broad vocabulary for cancer and skin toxicities, but only one term for intervention: “foot soak*” (not “foot bath*”, “balneology”, “hydrotherapy”), restriction on rcts was adequate and transparent 4. - |
|  | Acetyl-L-carnitine | Prevention of neurotoxicity: “insufficient evidence to formulate a recommendation” (p.13)  “The use of Acetyl-L-carnitine to prevent neurotoxicity of cancer treatment is not recommended outside the context of clinical research  (weak recommendation).” (p.52) | 1. Yes: Search strategy is presented in appendix 2. Yes: Medline, PreMedline, Embase, Central 3. Yes: Broad vocabulary on neuropathy and intervention of Acetyl-L-carnitine; restriction on rcts was adequate and transparent 4. - |
|  | Cannabinoids | Prevention of nausea and vomiting: “Cannabinoids are not recommended to treat nausea and vomiting  associated with chemotherapy or radiotherapy (weak recommendation).” (p.78) | 1. Yes: Search strategy is presented in appendix 2. Yes: Medline, Embase 3. Yes: Broad vocabulary on cancer, nausea, vomiting and cannabinoids; restriction on rcts was adequate and transparent 4. All studies (rcts and srs) are presented in detail in evidence tables |
|  | Nutritional supplements | Diarrhoea: “Nutritional supplements are not recommended to prevent diarrhoea in patients undergoing pelvic radiotherapy (weak recommendation).” (p.82) | 1. Yes: Search strategy is presented in appendix 2. Yes: Medline, Embase 3. Partly: Broad vocabulary for cancer, only one Mesh-Term for Diarrhea on Medline and only one Mesh-Term for the following interventions: vitamins, formulated food, dietary supplements, minerals, fortified food; 4. Yes: All relevant studies are presented in detail in evidence tables |
|  | Enzyme | Prevention of cardiotoxicity: “insufficient evidence to formulate a recommendation” (p. 13)  “The use of co-enzyme q10 to prevent cardiac toxicity of cancer  treatment is not recommended outside the context of clinical research  (weak recommendation).” (p. 83) | 1. Yes: Search strategy is presented in appendix 2. Yes: Medline, PreMedline, Embase, Central 3. Yes: Broad vocabulary for cardiac toxicities and various terms for the intervention co-enzymes; restriction on rcts adequate and transparent 4. - |
| Follow-up Care and Psychosocial Needs of Survivors of Prostate Cancer- PEBC, CCO - 2015 | Diet | “No diet plan can be recommended because no diet plan or food supplement has been associated with improved cancer outcomes.” (p. 14, Recommendation 6) | 1. Yes: Search string is presented in the appendix 2. Yes: Medline and Embase 3. Yes: Broad vocabulary on prostate cancer follow-up care, restrictions on predefined outcomes (e.g. fatigue, urinary dysfunction, quality of life) 4. Partly: Only basic information on the RCT is presented in an evidence table, indicating sample size, primary treatment, but no information on age, gender, exact outcomes and risk of bias. For the SR only a quality assessment is presented, but no information on included studies. |
| Chemoprevention of Lung Cancer – CHEST- 2013 | Beta- carotene | “For individuals with a greater than  20 pack year history of smoking or with a history of lung cancer, the use of β-carotene supplementation is not recommended for primary, secondary,  or tertiary chemoprevention of lung cancer (Grade 1A) .  Remarks: The dose of b carotene used in these studies was 20-30 mg per day or 50 mg every other day.” (p. e47S) | 1. No: Search strategy and results should be available on request, but there was no reaction on our request. In the mentioned methodological paper (Lewis 2013) only general information about the guideline development process are presented, but no details on the concrete search strategy 2. No: Only Medline 3. – 4. No: Studies are not presented in detail |
|  | Vitamin E, N-acetylcysteine | “For individuals at risk for lung cancer  and for patients with a history of lung cancer, the use of vitamin E, retinoids, and N-acetylcysteine and isotretinoin is not recommended for primary, secondary, or tertiary prevention  of lung cancer (Grade 1A) .” (p. e47S) | Same as above |
|  | Selenium | “In individuals with a history of early stage non-small cell lung cancer (NSCLC), the use of selenium as a tertiary chemopreventive agent of lung cancer is not recommended (Grade 1B).” (p. e48S) | Same as above |
|  | Tea extract  (metformin) | “In individuals at risk for lung cancer, the use of tea extract, or metformin is not suggested for primary, secondary or tertiary prevention of lung cancer (outside of the setting of a well-designed clinical trial) (Grade 2C).” (p. e41S) | Same as above |
| Management of Chronic Pain in Survivors of Adult Cancers –ASCO- 2016 | Acupuncture  Guided imagery  Massage  Mindfulness  Music  Relaxation | “Clinicians may prescribe directly or refer patients to other professionals to provide the interventions outlined in Table 4 to mitigate chronic pain or improve pain-related outcomes in cancer survivors. These interventions must take into consideration pre-existing diagnoses and comorbidities…  [Table 4 contains:]   - massage, acupuncture, music: strength of recommendation is weak - mindfulness, relaxation, guided imagery: strength of recommendation is moderate.” (p. 3, 10) | 1. Yes: Search string is presented in detail in data supplement 2. No: Only Pubmed 3. Partly: Narrow vocabulary, search terms as exercise, massage, yoga were used without any variances 4. Yes: All studies are presented in detail in data supplement online |
| Symptom Management in Patients With  Lung Cancer – CHEST- 2013 | Acupuncture  Aromatherapy  Healing/ Therapeutic Touch  Massage  Music  Reflexology  Reiki | “Acupuncture: A 2011 Cochrane review considered the evidence for acupuncture as a treatment of cancer pain. Three RCTs, including one of high quality, found positive results in favor of acupuncture when compared with placebo procedures or medication,  but problems with blinding and other methodologic issues led the authors to conclude that no recommendation could be made.” (p. 12)  “Aromatherapy and Massage: A systematic review was identified examining the role of aromatherapy and massage in cancer pain. There is evidence for a beneficial effect of massage on anxiety, although it is unclear whether aromatherapy has any additional effect. There is conflicting evidence for any impact of massage on physical symptoms, with three trials (117 participants) finding a reduction in pain. There is insufficient evidence upon which to base any recommendation.” (p. 12)  “Reflexology, Reiki, and Healing/ Therapeutic Touch: Partner-administered reflexology reduced pain intensity in patients with cancer in an RCT (n= 86). A systematic review of 66 studies investigating the  role of so-called biofield therapies (Reiki, healing touch, therapeutic touch) found moderate evidence for the reduction in pain intensity in patients with cancer. These studies suggest that touch therapies may have a role in treating cancer pain, and there is a need for further research.” (p. 12)  “Music: A Cochrane review of music for the treatment of all types of pain contained only one English-language study examining its effect in cancer  pain, involving 15 patients. There is insufficient evidence upon which to base a recommendation.” (p. 12) | 1. No: No search string is provided. In the referred methodological arcticle of Lewis 2013 there is only general information about the process 2. Yes: Medline, Google Scholar, CINAHL, PsycInfo, Cochrane, Embase, Web of Science 3. No: There is no information about the search string 4. No: None of the mentioned studies are presented in detail. |
| Antiemetics- ASCO- 2017 | Acupuncture/ Acupressure  Complementary/ alternative therapies  Ginger | “Complementary and alternative therapies: Evidence remains insufficient for a recommendation for or against the use of ginger, acupuncture/ acupressure, and other complementary or alternative therapies for the prevention of nausea and vomiting in patients with cancer.” (p.3) | 1. Yes: Search string is presented in the supplement 2. Yes: PubMed, Cochrane Library 3. Partly: Narrow vocabulary on “complementary medicine” 4. Yes: All studies are presented in detail in evidence tables in the supplement |
| Breast Cancer Survivorship Care Guideline- ACS/ASCO -2016 | Acupuncture | “It is recommended that primary care clinicians […] should offer one or more of the following interventions based on clinical indication: acupuncture, physical activity, and referral for physical therapy or rehabilitation (LOE 5 III).” (p. 48) | 1. Partly: A general search strategy is presented in the article, but the links of the terms are not exactly comprehensible 2. No: Only Pubmed 3. No: Search vocabulary is (according to the research question of the guideline) restricted to cancer survivors. No concrete vocabulary on acupuncture is included, only terms like “health promotion”, “psychosocial care” or “palliative care” 4. No: None of the mentioned studies is presented in detail in evidence tables. |
| **Weitere LL, die wir schon gefunden hatten** |  |  |  |
| Clinical Practice Guidelines on the Evidence-Based Use of  Integrative Therapies During and After Breast Cancer  Treatment – ACS- 2017 | Acetyl-L-Carnitine  Acupunture  Acupressure  Aloe vera  Ginger  Glutamine  Hypnosis  Massage  Meditation  Music therapy  Relaxation  Soy  Stress Management  Yoga | „Aloe vera and hyaluronic acid cream should not be recommended for improving acute radiation skin reaction.” Grade D  “Meditation is recommended for reducing anxiety.” Grade A  “Music therapy is recommended for reducing anxiety.” Grade B  “Stress management is recommended for reducing anxiety during treatment, but longer group programs are likely better than self-administered home programs or shorter programs.” Grade  B  “Yoga is recommended for reducing anxiety.” Grade B  “Acupuncture, massage, and relaxation can be considered for reducing anxiety.” Grade C  “Acupressure can be considered as an addition to antiemetics drugs to control nausea and vomiting during chemotherapy.” Grade B  “Electroacupuncture can be considered as an addition to antiemetics drugs to control vomiting during chemotherapy.” Grade B  “Ginger and relaxation can be considered as additions to antiemetic drugs to control nausea and vomiting during chemotherapy.” Grade C  “Glutamine should not be recommended for improving nausea and vomiting during chemotherapy.” Grade D  “Meditation, particularly MBSR, is recommended for treating mood disturbance and depressive  symptoms.” Grade A  “Relaxation is recommended for improving mood disturbance and depressive symptoms.” Grade A  “Yoga is recommended for improving mood and depressive symptoms.” Grade B  “Massage is recommended for improving mood disturbance.” Grade B  “Music therapy is recommended for improving mood.” Grade B  “Acupuncture, healing touch, and stress management can be considered for improving mood disturbance and depressive symptoms.” Grade C  “Hypnosis and ginseng can be considered for improving fatigue during treatment.” Grade C  “Acupuncture and yoga can be considered for improving post-treatment fatigue.” C  “Acetyl-L-carnitine and guarana should not be recommended for improving fatigue during treatment.” D  Acetyl-L-carnitine is not recommended for the prevention of chemotherapy-induced peripheral  neuropathy in patients with BC due to potential harm.” Grade H  “Acupuncture, healing touch, hypnosis, and music therapy can be considered for the management of pain.” Grade C  “Meditation is recommended for improving quality of life.” Grade A  “Yoga is recommended for improving quality of life.” Grade B  “Acupuncture, mistletoe, qigong, reflexology and stress management can be considered for improving quality of life.” Grade C  “Gentle yoga can be considered for improving sleep.” Grade C  “Acupuncture can be considered for improving hot flashes.” Grade C  “Soy is not recommended for hot flashes in patients with Breast Cancer due to lack of effect.” Grade D | 1. Partly: Search string is only presented in detail for Medline in an older version of the guideline “Clinical Practice Guidelines on the Use of Integrative Therapies” Greenlee 2014 (see below) 2. Yes: Embase, Medline, PsycInfo, Cinahl 3. Partly: Very broad vocabulary for cancer and outcomes, but search terms for study type (RCTs) is very restricted 4. Partly: Studies are presented in evidence tables in the supplement, but no concrete data on outcomes is made |
| Complementary Therapies and Integrative  Medicine in Lung Cancer – CHEST- 2013 | Mind-body | “In lung cancer patients experiencing the symptoms, mind-body modalities are suggested as part of a multidisciplinary approach to reduce  anxiety, mood disturbance, sleep disturbance, and improve QOL (Grade 2B). (p.7)  “In lung cancer patients experiencing the symptoms, mind-body modalities are suggested as part of a multidisciplinary approach to reduce  acute or chronic pain” (Grade 2B) . (p. 7.)  “In lung cancer patients experiencing the symptoms, mind-body modalities are suggested as part of a multidisciplinary approach to reduce anticipatory chemotherapy-induced nausea and vomiting” (Grade 2B). (p.7.)  “In lung cancer patients experiencing  the symptoms, yoga, a movement-based mindbody modality is suggested as part of a multidisciplinary approach to reduce fatigue and sleep disturbance while improving mood and QOL” (Grade 2B). (p. 7.) | 1. Partly: Search terms are presented in the article, but no detailed search string 2. Yes: MEDLINE, PubMed, and Web of Science 3. No: the search vocabulary was restricted on the outcomes of anxiety, dyspnea, fatigue, depression, but recommendations were also made for mood disturbance, sleep disturbance, quality of life, chronic pain, nausea, vomiting 4. Yes: All studies are presented in the supplement in detail with rating of study quality |
|  | Massage | “In lung cancer patients whose anxiety  or pain is not adequately controlled by usual care, addition of massage therapy performed by trained professionals is suggested as part of a multi-modality cancer supportive care program  (Grade 2B)”. (p. 8) | 1. Partly: Search terms are presented in the article 2. No: Only Pubmed 3. Yes: Search vocabulary was adequate and limitation to meta-analyses and reviews seems adequate, since two systematic reviews including 12-14 studies were found 4. Yes: All studies are presented in the supplement in detail with rating of study quality |
|  | Acupuncture | “In patients having nausea and vomiting from either chemotherapy or radiation therapy, acupuncture or related techniques is suggested as an adjunct treatment option” (Grade 2B). (p. 10)  “In patients with cancer related pain and peripheral neuropathy, acupuncture is suggested as an adjunct treatment in patients with inadequate control of symptoms” (Grade 2C). (p. 10) | 1. Partly: Search terms are presented in the article 2. Yes: Medline, PubMed, Web of Science 3. Yes: All restrictions of search string were adopted to concerning recommendations 4. Yes: All studies are presented in the supplement in detail with rating of study quality |
|  | Nutrition | “In patients undergoing treatment of lung cancer who have experienced weight loss, the addition of high calorie and protein supplements (1.5 kcal/mL) as a nutritional adjunct is suggested  to achieve weight stabilization” (Grade 2C) . (p. 12)  “In patients with lung cancer who have  sarcopenia oral nutritional supplementation with n-3 fatty acids is suggested in order to improve the nutritional status” (Grade 2C). (p. 12) | 1. Partly: Search terms are presented in the article 2. Yes: Medline and PubMed 3. Partly: Vocabulary for nutrition was only “nutrition”, no specific other terms of nutrition types 4. No: None of the studies are presented in detail. In the text is a referral to the guideline “Chemoprevention of Lung Cancer” from CHEST 2013, but there are no evidence tables also (for detailed information about this guideline see above) |
| **Statements and Recommendations relying on adaptions of other guidelines** | | | |
| Stomach cancer [Magenkarzinom] – GGPO – February 2012 | Nutrition | “Before major tumor resections in the upper GI area, patients should take preoperatively immunomodulatory drinking solutions containing arginine, omega-3 fatty acids and ribonucleotides even without signs of malnutrition.” (118: p. 126) | Adaption of ESPEN-Guidelines (Weimann 2006) was performed.  Evaluation of ESPEN- Guidelines:   1. Partly: General information about search strategy is presented (Schütz 2006). According to what is written an “adapted” literature search of the German guidelines of the German Society for Nutritional Medicine (DGEM) (Lochs 2003, 2004) was performed. 2. Yes: Medline, Embase, Pubmed, Cochrane 3. Partly: Restrictions were made to „humans“ and publication types of e.g. randomized controlled trials and systematic reviews. But since the concrete search strategy is not presented, no statement on adequacy of filters can be made. 4. No: None of the concerning studies is presented in detail in evidence tables (neather in Weimann 2006 nor in the underlying German guidelines of DGEM)   Evaluation of final adaption:   1. The statement was taken from the then 6-year-old ESPEN guideline. No additional search / update took place. 2. – 3. – 4. – |
| Breast cancer [Mammakarzinom] – GGPO – Version 4.1, September 2018 | Nutrition | “Patients should be advised to achieve and maintain a nutritional pattern that is high in vegetables, fruits, whole grains and legumes, low in saturated fats and limited in alcohol intake.” (4.81: p. 192) | Adaption of the American Cancer Society/ American Society of Clinical Oncology “Breast Cancer Survivorship Care Guideline” (Runowicz 2016) was performed, which was based on an older ACS Guideline (Rock 2012), which again was an adaption of a guideline by the US Department of Health and Human Services (2008) and an update of an older ACS Guideline (Doyle 2006), which was updated several times before (Brown 2003, Brown 2001, ACS Advisory Committee on Diet, Nutrition, and Cancer Prevention 1996, Weinhouse 1991).  Evaluation of first ACS Guidelines (Weinhouse 1991, ACS Advisory Committee on Diet, Nutrition, and Cancer Prevention 1996, Brown 2001, Brown 2003, Doyle 2006):   1. No: No search strategy is presented in any of these guidelines and their updates. 2. – 3. – 4. No: Mentioned studies are not presented in detail in evidence tables   Evaluation of the US Departments of Health and Human Services “Physical Activity Guidelines for Americans” (2008):   1. Yes: Search strategy is presented (Appendix F.1, F.2) 2. No: Only Medline 3. Yes: Broad vocabulary with Mesh- and text-terms for all cancer entities. 4. No: None of the mentioned studies is presented in detail in evidence tables. No assessment of study quality and risk of bias of included studies.   Evaluation of ACS “Nutrition and Physical Activity Guidelines for Cancer Survivors” (Rock 2012):   1. No: In general the guideline summarizes the findings from the report of the ACS (Doyle 2006). Although it is mentioned that new evidence has evolved since 2006, no search strategy is presented. Concerning physical activity the authors refer to the recommendation of the American College of Sports Medicine (ACSM) (Schmitz 2010) to follow the “Physical Activity Guidelines for Americans” (US Department of Health and Human Services 2008). 2. No: - 3. No: - 4. No: None of the mentioned studies is presented in detail in evidence tables.   Evaluation of ACS/ASCO “Breast Cancer Survivorship Care Guideline” (Runowicz 2016):   1. Yes: The statements on physical activity are mainly adapted from an older ACS/ASCO guideline (Rock 2012). A general search strategy is presented. 2. No: Only Pubmed. 3. No: Search vocabulary is (according to the research question of the guideline) restricted to cancer survivors. No concrete vocabulary on physical activity or nutrition is included, only terms like “health promotion”, “psychosocial care” or “palliative care” 4. No: None of the mentioned studies is presented in detail in evidence tables.   Evaluation of final adaption:   1. No additional update of the search took place. 5 further publications are mentioned, two of them are also guidelines, and among this is the ACS Guideline (Rock 2012). 2. - 3. -   No: A: No evaluation of the 2 further guidelines is presented and none of the 3 studies are presented in detail! B-F: None of the mentioned studies is presented in detail. |
| Supportive therapy [Supportive Therapie] – GGPO – Version 1.1, April 2017 | Vitamin D | “To prevent tumor-associated osteoporosis, daily vitamin D supplementation with 800 to 1,000 units of vitamin D3 should be given.” (10.54: p. 317) | Adaption of the guideline “OSTEOPOROSE in men over the age of 60 and in postmenopausal women” of the Dachverband der Deutschsprachigen wissenschaftlichen Osteologischen Gesellschaften e.V. 2014 (DVO 2014)  Evaluation of the adapted guideline (DVO 2014):   1. No: This guideline is an adaption of the former versions of the DVO guidelines, which was first developed in 2003. For this update a search of other guidelines, systematic reviews and meta-analysis was conducted. There is no concrete information on the process (which guidelines were chosen for which research questions). Besides the information, that the search strategy of the update used the term „osteoporosis“, there is no search strategy presented (report p. 4). 2. No: Only Medline and a specific selection of 4 journals on osteoporosis 3. Partly: Restrictions were made to systematic reviews and meta-analysis, no restrictions on cancer type. Since the concrete search strategy is not presented, no statement on adequacy of filters can be made. 4. No: None of the mentioned studies are presented in detail in evidence tables.   Evaluation of Adaption:   1. The adapted guideline applies only to postmenopausal woman and men over the age of 60 years. The original statement is: “The guideline group recommends drug supplementation with 800-1000 units of vitamin D3 daily in people at high risk of falling and / or fractures with low exposure to sunlight” (p. 148). No additional search/ update concerning vitamin D took place. 2. – 3. - 4. - |
| Prostate Cancer Survivorship Care Guideline- ASCO -2015 | Diet including Vitamin D | “Counsel survivors to achieve a dietary pattern that is high in fruits and vegetables and whole grains.  a. Consume a diet emphasizing micronutrient-rich and phytochemical-rich vegetables and fruits, low amounts of saturated fat, intake of at least 600 IU of vitamin D per day, and consuming adequate, but not excessive, amounts of  dietary sources of calcium (not to exceed 1,200 mg/d).  b. Refer survivors with nutrition-related challenges (eg, bowel problems that impact nutrient absorption) to a registered dietitian.” (p. 1079) | Adaption of the guideline “Prostate Cancer Survivorship Care Guidelines” of ACS (Skolarus 2014)  Evaluation of Skolarus 2014:   1. Partly: Only general search terms are presented in the article itself 2. No: Only Medline 3. No: Restrictions are incomprehensible 4. No: Studies are not described in detail, no evidence tables   Evaluation of final update:   1. No: Supplement not available 2. No: Only Medline 3. No: Supplement not available, according to the authors, the search strategy described in ASC guideline (Skolarus 2014) was adapted 4. No: Supplement not available |
| * Evaluation of statements and recommendations which are declared as evidence based  1. provided a search strategy or at least key words with comprehensive vocabulary?  2. searched at least 2 databases?  3. used appropriate restrictions to the research question (inclusion/exclusion criteria, publication type, study type)  4. described the included studies in adequate detail/ presented evidence tables? | | | |
